# Study on the mechanisms of compound Kushen injection for the treatment of gastric cancer based on network pharmacology

**DOI:** 10.1186/s12906-019-2787-y

**Published:** 2020-01-15

**Authors:** Wei Zhou, Jiarui Wu, Yingli Zhu, Ziqi Meng, Xinkui Liu, Shuyu Liu, Mengwei Ni, Shanshan Jia, Jingyuan Zhang, Siyu Guo

**Affiliations:** Department of Clinical Chinese Pharmacy, School of Chinese Materia Medica, Beijing University of Chinese Medicine, No. 11 of North Three-ring East Road, Chao Yang District, Beijing, China

**Keywords:** Compound Kushen injection, Gastric Cancer, Network pharmacology

## Abstract

**Background:**

As an effective prescription for gastric cancer (GC), Compound Kushen Injection (CKI) has been widely used even though few molecular mechanism analyses have been carried out.

**Methods:**

In this study, we identified 16 active ingredients and 60 GC target proteins. Then, we established a compound-predicted target network and a GC target protein-protein interaction (PPI) network by Cytoscape 3.5.1 and systematically analyzed the potential targets of CKI for the treatment of GC. Finally, molecular docking was applied to verify the key targets. In addition, we analyzed the mechanism of action of the predicted targets by Kyoto Encyclopedia of Genes and Genomes (KEGG) and Gene Ontology (GO) analyses.

**Results:**

The results showed that the potential targets, including CCND1, PIK3CA, AKT1, MAPK1, ERBB2, and MMP2, are the therapeutic targets of CKI for the treatment of GC. Functional enrichment analysis indicated that CKI has a therapeutic effect on GC by synergistically regulating some biological pathways, such as the cell cycle, pathways in cancer, the PI3K-AKT signaling pathway, the mTOR signaling pathway, and the FoxO signaling pathway. Moreover, molecular docking simulation indicated that the compounds had good binding activity to PIK3CA, AKT1, MAPK1, ERBB2, and MMP2 in vivo.

**Conclusion:**

This research partially highlighted the molecular mechanism of CKI for the treatment of GC, which has great potential in the identification of the effective compounds in CKI and biomarkers to treat GC.

## Background

Gastric cancer (GC) is one of the most common malignant tumors, and its mortality rate ranks third in the world, making it the fifth most frequently diagnosed cancer according to the latest report of Global Cancer Statistics (GLOBOCAN 2018). The global incidence rate varies widely, with the highest incidence rates in East Asia and Eastern Europe, while North America, parts of Africa and Northern Europe have the lowest incidence [1]. Environmental factors, including *Helicobacter pylori* (*H. pylori*) infection, smoking, high salt intake, and other dietary factors, can increase the risk of GC [2]. GC is often diagnosed at an advanced stage, and surgery can possibly cure patients with GC; moreover, a multimodality approach can increase survival [3]. Combined in different forms and with different drugs, chemotherapy and radiotherapy are common methods for the treatment of GC, and chemotherapy shows the best supportive care in advanced GC [4]. However, the adverse effects of radiotherapy and chemotherapy frequently cause physical damage to patients, such as upper limb swelling on the affected side, decreased white blood cells and immunity, gastrointestinal reactions, bone marrow suppression, immune dysfunction and organ damage, causing tremendous pain to the patients [5, 6]. As an important part of complementary and alternative medicine, traditional Chinese medicine has been widely used in the clinical treatment of cancer in Asian countries, especially in China and Japan [7]. Compound Kushen Injection (CKI) consists of two herbs, Kushen (Radix Sophorae Flavescentis) and Baituling (Rhizoma Smilacis Glabrae). CKI is often used to treat many types of solid tumors, such as hepatic carcinoma, lung cancer and GC, and it is increasingly being used for cancer-related pain and has been used clinically in China for more than 15 years [8, 9]. Kushen has a long history of being used for the treatment of solid tumors, inflammation and other diseases, such as gynecological diseases [10]. Studies have found that Baituling can not only enhance the cytotoxicity of Kushen in CKI, but also activate immune-related pathways [11]. CKI mainly contains various anticancer ingredients such as matrine and oxymatrine, which can inhibit tumor cell growth, induce the apoptosis of cancer cells, and resist cancer cell metastasis, and is unsusceptible to multidrug resistance and protective against overactive immune responses in the body [12]. Research has shown that the matrine in CKI can inhibit cell proliferation and induce apoptosis in gastric carcinoma cells (SGC-7901) [13]. CKI can limit mouse sarcoma growth by reducing the phosphorylation of ERK and AKT kinases and BAD. CKI can also block TRPV1 signaling by inhibiting TRPV1-mediated capsaicin-induced ERK phosphorylation and reducing tumor-induced proinflammatory cytokine production, indirectly limiting cancer pain by reducing tumor growth [14]. The chemical composition of traditional Chinese medicine (TCM) compound is complex, and they are usually prepared by the synergy of multiple Chinese medicines and multiple targets to exert pharmacodynamic effects [15]. The network pharmacology “disease-gene-target-drug” research model is very consistent with the synergistic effect of the TCM “multicomponent, multichannel, multitarget” model, which more effectively reveals the relationship between TCM compound preparation and disease [16]. As a new field, network pharmacology provides a deeper insight into the basic mechanisms of TCM theory by using systems biology, highly integrated data analysis strategies and visualizations of the interpretations [17]. TCM network pharmacology has the role of discovering biologically active compounds and elucidating the mechanisms of action of TCM formulas, with the significance of accelerating the discovery of TCM and improving current drug discovery strategies [18]. Besides, molecular docking methods were used to assess the binding of components in CKI to key targets to confirm the drug’s effect on the target [19].

**Fig. 1:**
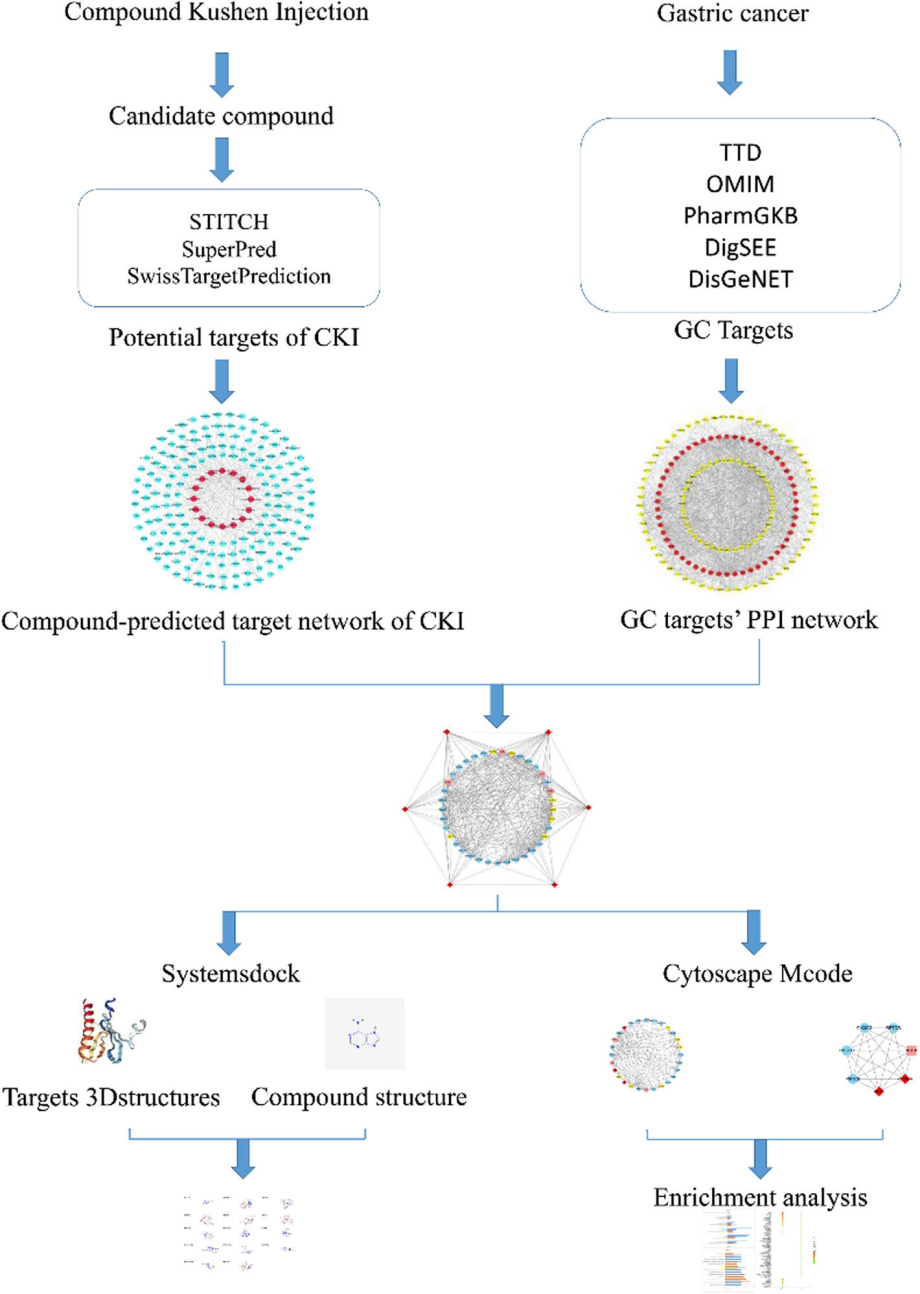
Workflow for CKI in treating GC

Previous studies have confirmed the effectiveness of CKI in the treatment of GC [20, 21] but did not explore the molecular biology. To better analyze and predict the molecular mechanism of CKI in the treatment of GC, this study adopted the network pharmacology and molecular docking method. The therapeutic target prediction of active ingredients and drug target network analysis were systematically carried out and may provide a reference for the basic experimental research and clinical rational application of CKI in the treatment of GC. The detailed workflow of the network pharmacology-based study of CKI is shown in Fig. 1.

>

## Methods

### Collection of target proteins related to GC

The Therapeutic Target Database (TTD) [22] (http://bidd.nus.edu.sg/group/ttd/ttd.asp) is a database that allows users to search for targets, biomarkers, and drugs related to various disease conditions. Online Mendelian Inheritance in Man (OMIM) [23] (http://www.omim.org/) is an online database of continuously updated human genes and genetic diseases. Pharmacogenomics Knowledge Base (PharmGKB) [24] (http://www.pharmgkb.org) is a database of high-quality pharmacogenomic data that provides information on genetic variation, pharmacokinetics, and pharmacodynamic pathways, as well as data on the effects of mutations on drug-related phenotypes. The disease gene search engine with evidence sentences (DigSEE) [25] (http://210.107.182.61/geneSearch/) database can directly query the links between diseases, genes and biological processes. DisGeNET [26] (http://www.disgenet.org) is one of the most comprehensive genetic disease association databases. The above databases were searched for “gastric cancer” as a keyword to collect proteins related to GC.

### Collection of CKI active ingredients

Following a literature search [27], the 23 active ingredients contained in CKI were selected for research (Additional file 1: Table S1), and the three-dimensional structure data of 16 active ingredients were searched and derived from the PubChem database [28].

### Prediction of potential targets of CKI

The 3D chemical structure data of 16 active ingredients were imported into the Search Tool for Interactions of Chemicals (STITCH), SuperPred, and SwissTargetPrediction databases for retrieval. STITCH shows the link between proteins and small molecules in biological processes, visualizing them in a network, and provides information such as the strength of their association; it can also be used to search for potential targets for compounds [29]. SuperPred uses chemical similarity principles to predict possible biological targets of drugs and demonstrates the interaction between compounds and predicted targets [30]. SwissTargetPrediction performs a combination of similarity measurements based on known 2D and 3D chemical structures to predict the corresponding potential bioactive targets [31]. The predicted multiple target information of the compounds and the obtained information were introduced into Cytoscape 3.5.1 (http://www.cytoscape.org/) to obtain a compound-predicted target network map. Cytoscape is bioinformatics analysis software that visualizes biological pathways and intermolecular interaction networks. It provides a basic set of data integration, analysis and visualization capabilities for complex network analyses [32].

### Construction of related protein interaction networks

Search Tool for the Retrieval of Interacting Genes/Proteins (STRING) 10.5 (https://string-db.org/) is a database of known and predicted protein interactions that contains direct and indirect associations of proteins. The number and quality of interacting proteins can be set according to their confidence settings, and it has a score for each protein interaction. The higher the score is, the higher the confidence of the protein interaction [33]. The proteins related to GC collected by the databases and the proteins predicted according to the 3D structure of the compound were input into the STRING 10.5 database, and the species selection “*Homo sapiens*” was selected as the confidence data with a scoring value greater than 0.7. Then, the data were introduced into Cytoscape to construct a protein-protein interaction (PPI) network related to GC and a PPI network related to the predicted target of the compound.

### Network merge

The active components of CKI and the predicted target PPI networks were combined with the GC-PPI networks. The overlapping proteins in the two networks are likely to be potential targets for the treatment of GC by the active ingredients of CKI. A potential key target network for the CKI treatment of GC was constructed by Cytoscape, and the potential targets of the network were systematically analyzed.

### GO functional and KEGG pathway enrichment analysis

To illustrate the role of the key potential targets in gene function and signaling pathways, this study used the Database for Annotation, Visualization and Integrated Discovery (DAVID) v 6.8 [34] (https://david.ncifcrf.gov/) to perform GO functional enrichment analyses and KEGG pathway enrichment analyses on the potential key targets in the CKI network for the treatment of GC.

### Molecular docking simulation

The molecular docking of systemsDock [19] (http://systemsdock.unit.oist.jp/iddp/home/index) is a web server based on the docking program designed by AutoDock VINA with a high-precision scoring function. It has the characteristics of high precision and fast speed. The docking score for systemsDock is the negative logarithm of the experimental dissociation/inhibition constant (pKd/pKi), which directly indicates the binding strength.

## Results

### PPI network of GC targets

**Fig. 2:**
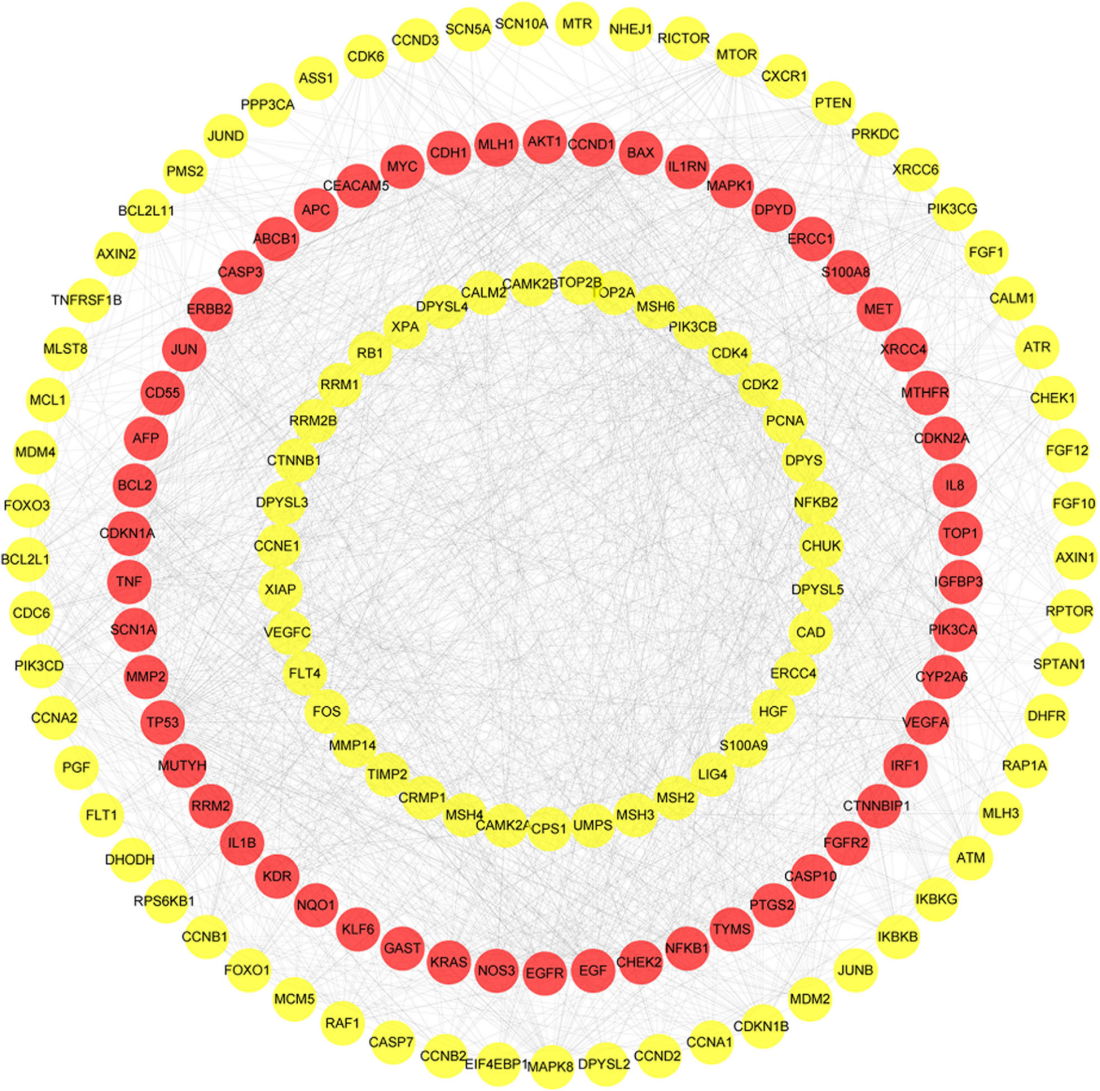
PPI network of GC targets (red nodes are disease-related targets and yellow nodes are 94 other related human targets)

A total of 60 target proteins (after removal of duplicates) related to GC were retrieved from the TTD, OMIM, PharmGKB, DigSEE, and DisGeNET databases (Additional file 2: Table S2). The PPI network was constructed in the STRING 10.5 database and included 154 nodes (60 disease-related nodes and 94 other related human target nodes), which constitute 1484 protein interactions (Fig. 2).

### Compound-**predicted** target network

**Fig. 3:**
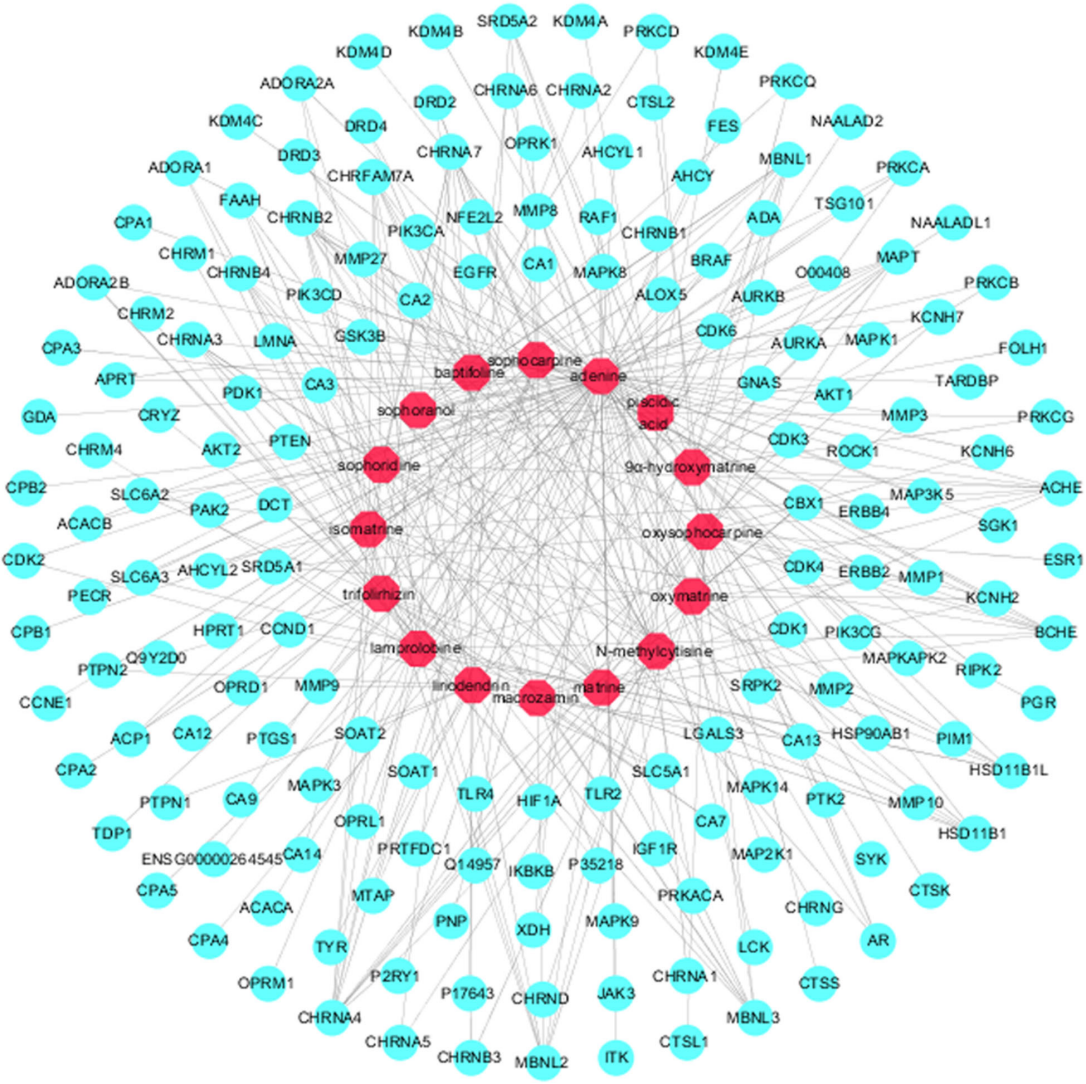
Compound-predicted target network of CKI (16 compound nodes are red, and 174 target nodes are blue-green)

Basic information on the 16 active ingredients in CKI is shown in Table 1. The active compound-predicted target network (Fig. 3) consists of 190 nodes (16 compound nodes and 174 target nodes) that constitute 331 active compound-predicted target linkages (Additional file 3: Table S3).

**Table 1:** Information on the active ingredients of CKI

PubChem CID	COMPOUND	STRUCTURE	PubChem CID	COMPOUND	STRUCTURE
15385684	9α-hydroxymatrine		87752	lamprolobine	
190	adenine		226371	liriodendrin	
621307	baptifoline		9576780	macrozamin	
5271984	isomatrine		91466	matrine	
115269	sophocarpine		670971	N-methylcytisine	
12442899	sophoranol		24864132	oxymatrine	
165549	sophoridine		24721085	oxysophocarpine	
442827	trifolirhizin		6710641	piscidic acid

### Potential key target network for the treatment of GC with CKI

**Fig. 4:**
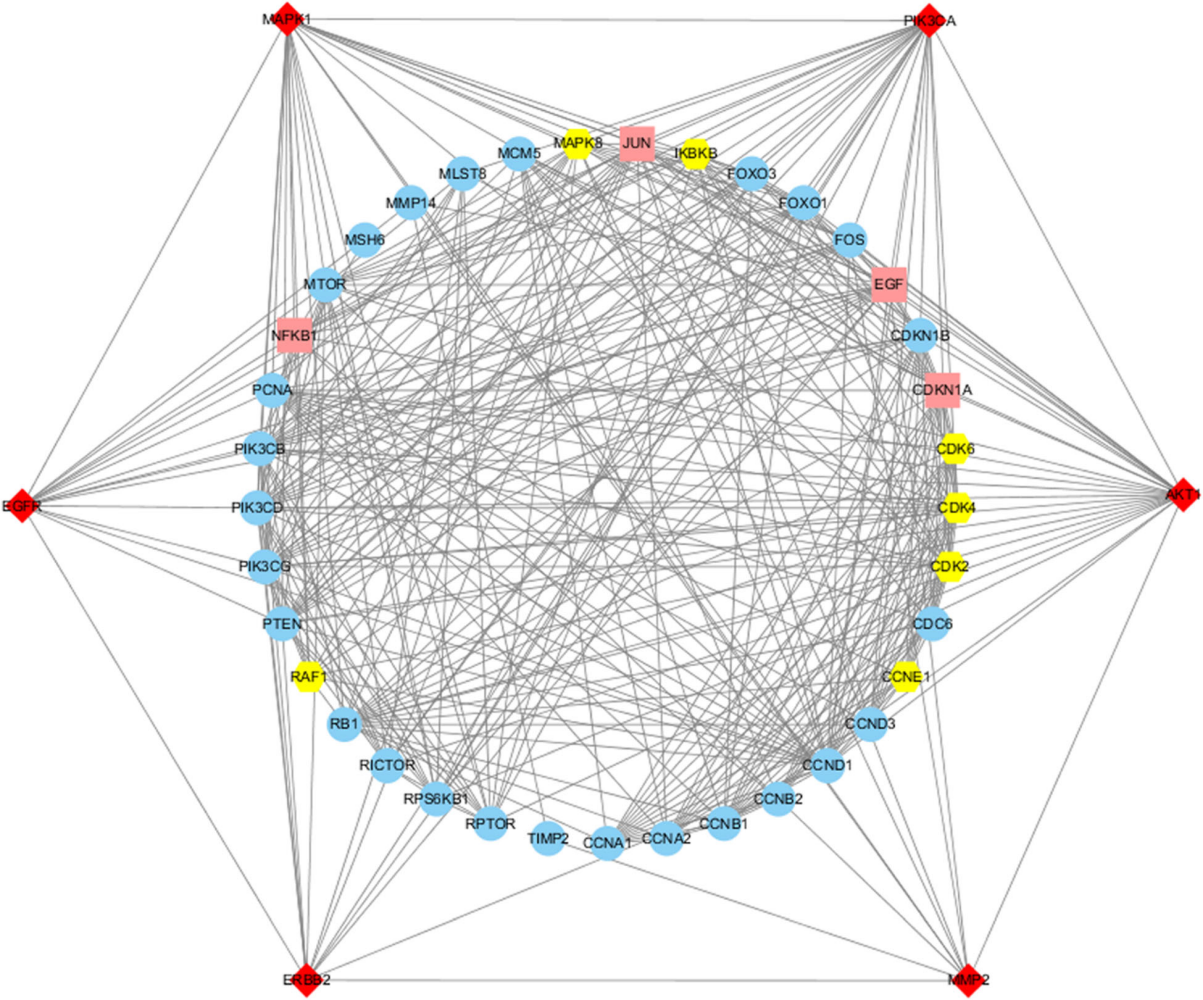
Potential key target network of CKI for the treatment of GC (red diamonds represent the same targets for GC and CKI components. Pink squares indicate the targets of the disease. Yellow hexagons represent the targets of the CKI active ingredients. Blue circles represent PPI-related human targets)

The CKI active compound-predicted target PPI network was combined with the GC-PPI network to remove proteins that did not intersect, and the potential targets of CKI for the treatment of GC were intuitively obtained. Essentially, the node connections in the network identify the same targets in the CKI active component prediction target PPI network as those found in the GC-PPI network, that is, the potential targets of the CKI active ingredient for treating GC. Using Cytoscape software to obtain the potential target degree, the greater the degree is, the greater the possibility that the CKI active ingredients will act on GC through the targets. The potential target network of CKI for the treatment of GC is shown in the Fig. 4 and includes 43 targets (6 targets for disease and active ingredients, 4 disease-related targets, 7 active-related targets, and 26 other related human targets) and 358 potential links. The targets of the GC-CKI active component potential target network have an average degree of 16.65. Among the targets, the degree of 22 targets is greater than or equal to 17, indicating that they are likely to be vital potential targets for the treatment of GC with the active ingredients in CKI.

### Molecular docking verification

**Fig. 5:**
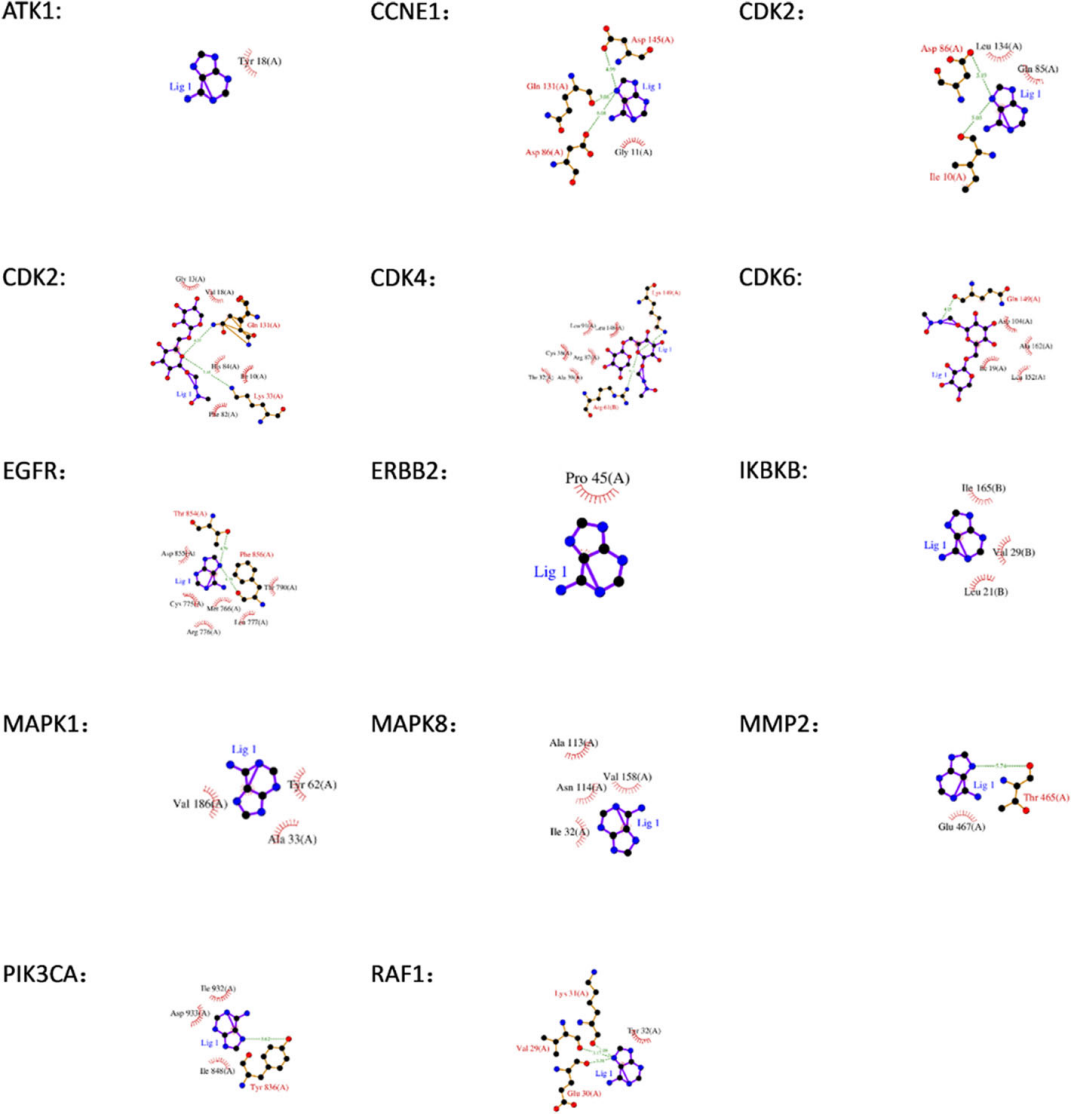
Detailed target-compound interactions of the docking simulation

Fourteen of the potential targets were directly related to the CKI active components, and these 14 targets were imported into the Protein Data Bank (PDB) (https://www.rcsb.org/) [35] database to find their 3D structure. The 3D structure of CCND1 could not be obtained, but the remaining 13 protein structures were available. The 3D structures of the remaining 13 targets were introduced into systemsDock for molecular docking verification. Most of the docking scores were larger than 5, which showed that the proteins possessed good binding activity [36]. The docking results are shown in Fig. 5. The docking details are shown in Table 2. The protein structures were showed by Pymol [37].

**Table 2:** Information on molecular docking

No.	Protein Name	PDB ID	Protein structure	Test Compounds	Docking Scores (pKd/pKi)
1	AKT1	1UNQ		adenine	6.394
2	CCNE1	5L2W		adenine	5.443
3	CDK2	2R3I		adenine	5.669
4	CDK2	2R3I		macrozamin	5.922
5	CDK4	2 W96		macrozamin	5.153
6	CDK6	1BLX		macrozamin	6.431
7	EGFR	3W2S		adenine	5.935
8	ERBB2	2A91		adenine	6.403
9	IKBKB	4KIK		adenine	6.45
10	MAPK1	3O71		adenine	6.291
11	MAPK8	3PZE		adenine	5.754
12	MMP2	1GEN		adenine	6.4
13	PIK3CA	5DXT		adenine	6.326
14	RAF1	1C1Y		adenine	6.105

### GO functional and KEGG pathway enrichment analysis

**Fig. 6:**
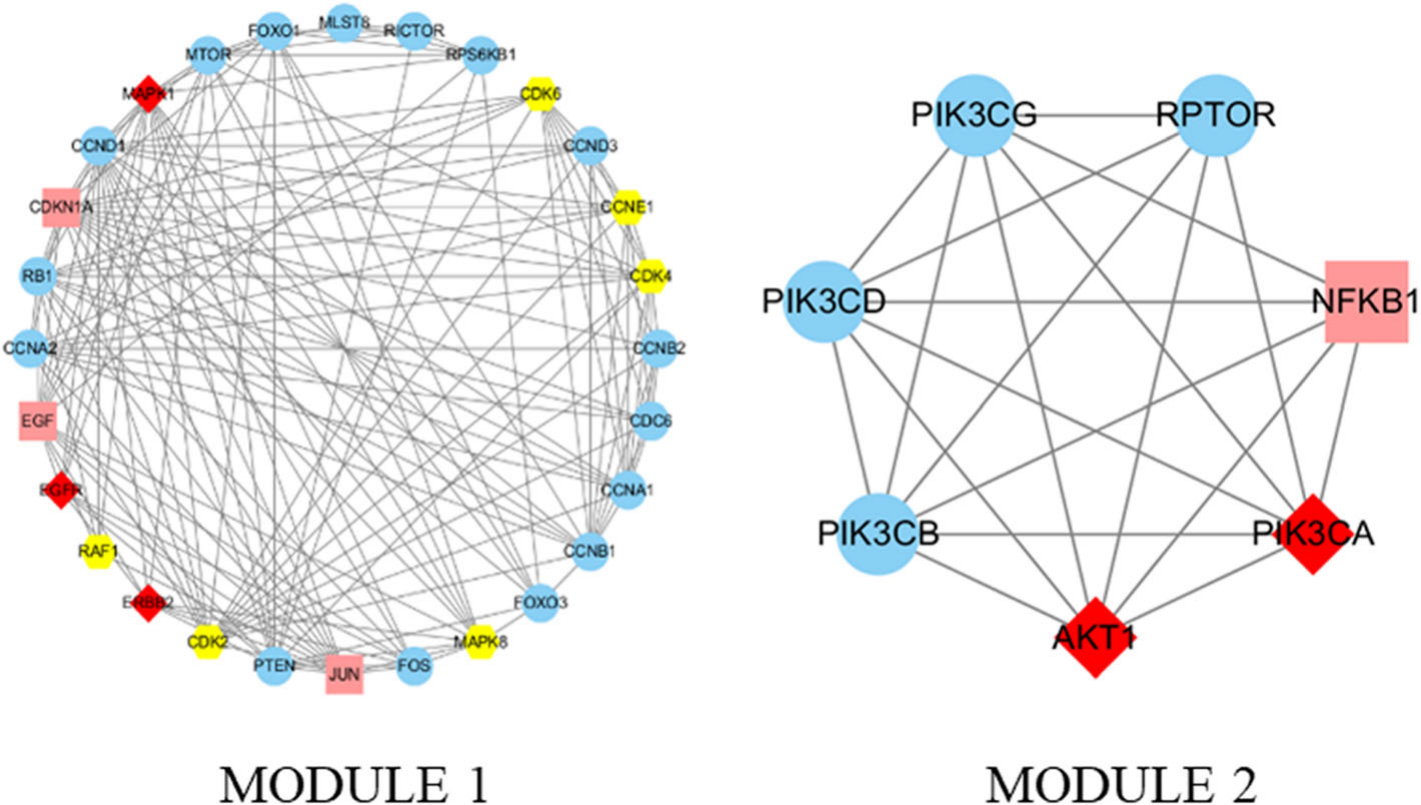
Module analysis infographic

**Fig. 7:**
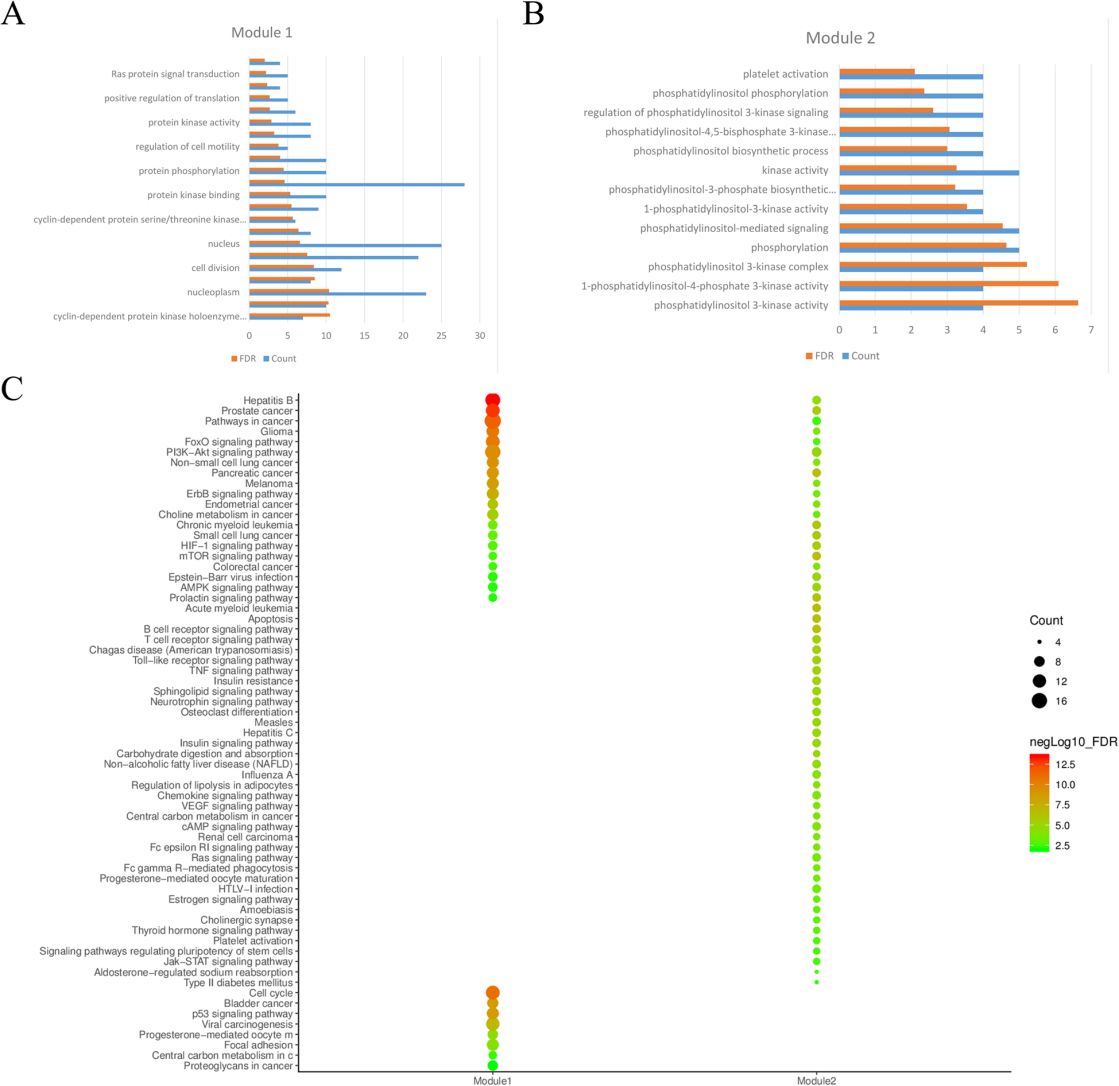
Graph of the enrichment analysis results (A. Graph of Module 1 GO analysis results; B. Graph of Module 2 GO analysis results; and C. Chart of KEGG enrichment analysis results)

**Fig. 8:**
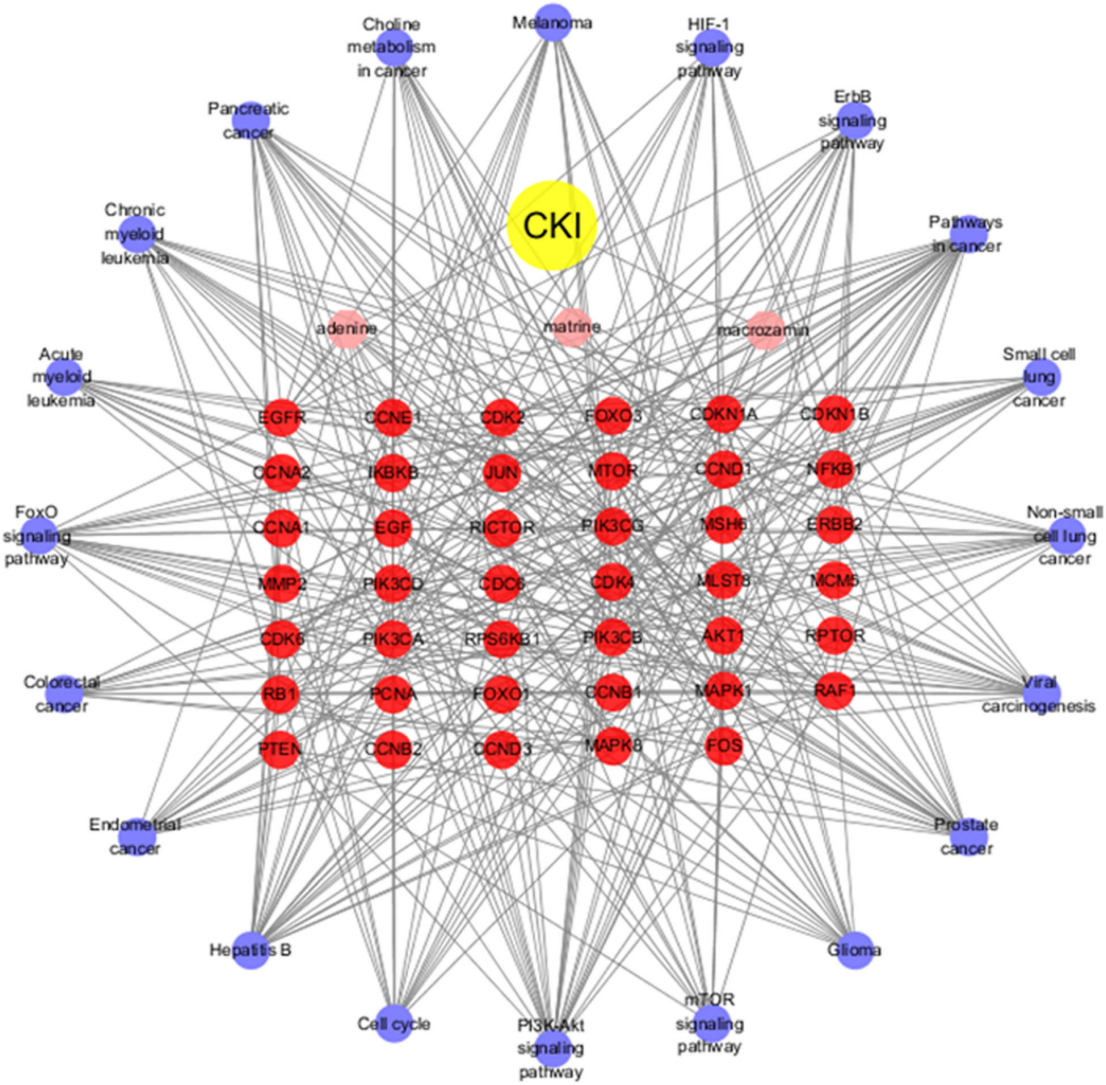
Drug-ingredient-target-pathway network. This network intuitively illustrates the interactions of the chemical components, predictive targets and pathways of CKI for the treatment of GC. (The yellow node represents CKI. The pink nodes represent the three active components in CKI. The red nodes represent the corresponding key targets. The purple nodes represent the target KEGG enrichment pathways)

Using the MCODE algorithm in Cytoscape for cluster analysis [38], two modules were obtained after clustering. Module 1 contained 28 nodes with 157 edges, and Module 2 contained 7 nodes with 20 edges (Fig. 6). Using the DAVID platform for GO functional enrichment analysis, the roles of the 43 target proteins involved in the Module 1 and Module 2 networks in gene function were studied. For the respective modules, 22 and 13 GO entries were determined based on the false discovery rate (FDR) and P values (*FDR < 0.01 and P < 0.01*). KEGG function enrichment analysis was performed by using the DAVID platform, and the roles of the 43 target proteins involved in the Module 1 and Module 2 networks in signaling pathways were studied. A total of 76 KEGG entries were determined based on the FDR and *P* values (*FDR < 0.01 and P < 0.01*) (Fig. 7). The overall mechanism prediction of the effect of CKI on the treatment of gastric cancer is shown in Fig. 8.

## Discussion

The incidence of GC is extremely high in many countries worldwide, and the incidence of GC is much higher in China than in many other countries [39]. CKI has been clinically used in China for over 15 years to treat various types of solid tumors, including GC. This study constructed a PPI network related to GC, a CKI active component-predicted target network, and a potential target network of CKI for the treatment of GC, and then systematically analyzed the mechanism of action of CKI in the treatment of GC. The molecular docking method was used to verify the correspondence between the target and the component, and the good docking score reflects the effectiveness of the component.

The potential target network of CKI for the treatment of GC contained a total of 43 targets, sorted into CCND1, PIK3CA, PIK3CG, PIK3CD, AKT1, PIK3CB, CDKN1B, EGF, CDKN1A, CDK2, MAPK1, JUN, CDK4, PTEN, MTOR, RB1, MAPK8, FoxO1, CCNB1, FOS, PCNA, EGFR, FoxO3, NFKB1, CDK6, RPTOR, IKBKB, CCNA1, RPS6KB1, CCNA2, ERBB2, CDC6, CCND3, CCNE1, RAF1, MCM5, RICTOR, CCNB2, MMP2, MLST8, MMP14, TIMP2 and MSH6 according to the their degree value, and the degree of the first 21 targets is higher than the average. In addition, among the 43 targets, the following were targets for both GC and CKI: CCND1, PIK3CA, AKT1, MAPK1, ERBB2 and MMP2. These targets may be key targets of CKI for the treatment of GC.

Cyclin D1 (CCND1) is an important cell cycle regulatory protein that primarily controls the transition of proteins from G1 to S in the cell cycle. The overexpression of CCND1 can cause a variety of tumors, increasing tumor cell proliferation, and affecting the prognosis of cancer patients [40]. Kaplan-Meier analysis indicated that an overexpression of CCND1 affected the survival of GC patients. Existing studies have shown that forcing the expression of miR-33a can inhibit the overexpression of CCND1 and inhibit the proliferation of GC cells [41, 42]. A study showed that CCND1 in MCF-7 breast cancer cells using CKI was significantly down-regulated compared with untreated cells [43].Therefore, CCND1 is predicted to be a significant target of CKI for the treatment of GC. Phosphatidylinositol-3-kinase catalytic subunit (PIK3CA) is the third most frequently mutated gene in GC [44, 45]. Clinical trial studies have shown that PIK3CA mutations are associated with increased tumor aggressiveness, especially in locoregional disease, and Akt activation in GC [46]. AKT1, also known as protein kinase B (PKB), is a serine/threonine protein kinase. Existing experiments have demonstrated that the activation of AKT inhibits the abundance of p53, leading to a decrease in the transcription of miR-365, an upregulation of CCND1 and cdc25A, and the promotion of gastric cell proliferation [47]. Therefore, it is speculated that both PIK3CA and AKT1 may be targets of GC treated with CKI. Furthermore, GC can be treated by inhibiting the expression of PIK3CA and AKT1, leading to an inhibition of the growth and proliferation of GC cells. Mitogen-activated protein kinases (MAPKs) are a group of silk/threonine kinases that are activated by a variety of signals. Mitogen-activated protein kinase 1 (MAPK1), also known as extracellular signal-regulated kinase 2 (ERK2), is involved in the regulation of cell differentiation and proliferation. MAPK1 is the core of the ras-MAPK signaling pathway. Activated MAPK1 transmits signals to the nucleus, phosphorylates and activates various nuclear transcription factors, and promotes and regulates some gene expression related to cell growth and differentiation. Abnormalities in MAPK1 can lead to the development of multiple cancers [48]. It has been demonstrated that MAPK1 can be mediated by multiple microRNAs to reduce the proliferation and metastasis of GC cells [49, 50]. Transcriptome analysis of CKI-treated MDA-MB-231 cells revealed that PIK3CA, AKT1, and MAPK1 all affect cell migration, which may be related to the role of CKI in the treatment of cancer [51]. The epidermal growth factor-related protein (ErbB) family of receptor tyrosine kinases plays an important role in epithelial cell development. The overexpression of human epidermal growth factor receptor 2 (ERBB2) promotes the differentiation and proliferation of cancer cells, which is recognized as one of the key causes of GC and seriously affects the prognosis of GC patients [52, 53]. At present, there is no in vivo or in vitro experiment to confirm that CKI achieves the therapeutic effect of GC by interfering with ERBB2, which is an important potential direction for future research. MMPs are a family of highly conserved endopeptidases that participate in a variety of physiological and pathological processes, such as inflammatory response, tissue ischemia and hypoxia injury, tissue fibrosis, neovascularization, and tumor adhesion, invasion, and metastasis [54]. MMP2 plays an important role in the invasion and metastasis of GC and can promote tumor invasion and metastasis by degrading the extracellular matrix, promoting neovascularization, and regulating cell adhesion [55]. Li et al. [9] found that various components of CKI can act on MMP2 and confirmed by Western blotting that CKI significantly downregulated MMP2.

The clustering analysis was carried out by using the MCODE algorithm in Cytoscape, and two modules were obtained by clustering. Among them, Module 1 had a higher number of nodes, edges, and scores, which may be the main functional module. To further understand the overall process of CKI in the treatment of GC, the GO and KEGG enrichment functions of the two modules obtained in this study were analyzed.

In the GO functional enrichment analysis, the target proteins were divided into different functional modules. Module 1 had the most biological process-related entries, including the regulation of cell mitosis, cell proliferation and transcription, protein phosphorylation, and protein signaling. Module 2 contained 13 related items, showing a strong correlation with phosphatidylinositol and its kinase-mediated signaling.

In the KEGG pathway enrichment analysis, the different modules obtained 76 related items by FDR < 0.01 screening analysis. The 76 related pathways included cancer pathways and disease pathways, most of which were related to signaling pathways, such as the PI3K-AKT signaling pathway, FoxO signaling pathway, mTOR signaling pathway, ErbB signaling pathway, AMPK signaling pathway, and HIF-1 signaling pathway. Therefore, the predicted mechanism of CKI for the treatment of GC is related to these pathways.

PI3K signaling is a critical regulator of many important cellular processes, including cell growth, metabolism, survival, metastasis, and resistance to chemotherapy. PI3K and AKT proteins are significantly overexpressed in tumor tissues. AKT is a downstream effector of PTEN/PI3K, and the inactivation of PTEN will result in an abnormal activation of AKT. p-AKT is an activated form of AKT that plays an important role in cell proliferation, differentiation and survival. Studies have found that it is abnormally activated in various tumors, such as lung cancer, breast cancer, and colorectal cancer. mTOR is located downstream of the PI3K/AKT/mTOR signaling pathway. After activation, it mediates important downstream signaling molecules and affects cell proliferation and inhibits apoptosis. High mTOR expression is found in many malignant tumors [38, 56, 57]. The epidermal growth factor receptor (EGFR) family plays an important role in growth and development, and its alteration is associated with a variety of conditions, including cancer. This receptor family mediates cell proliferation and survival through the MAPK and PI3K/KT signaling pathways. Distortion of the ErbB3/PI3 kinase pathway is common in GC, especially in diffuse tumors, leading to an excessive activation of the PI3K-AKT signaling pathway [58]. Based on a specific genotype, the FoxO family is a transcription factor that plays an important role in apoptosis, cell cycle arrest, DNA repair, and oxidative stress by regulating downstream target gene transcription levels. The most important pathway for the interaction with FoxO in different types of cancer is the PI3K-AKT pathway [59, 60]. In a current experiment, transcriptomic analysis of CKI-treated non-small cell lung cancer cell lines revealed that CKI does regulate ErbB, MAPK, PI3K/Akt and other pathways. Flow cytometry analysis confirmed that mTOR is located downstream of PI3K/Akt and is the main pathway for CKI to regulate cancer cells [61]. Therefore, these potential targets and pathways may be key for CKI treatment of GC.

The limitations of our study were as follows: Firstly, our study has some limitations because it focuses on genes that were obtained data in the network databases, without regard to gender, age, tumor classification, and staging. Secondly, our data research results need to be verified by corresponding experimental studies.

## Conclusion

In summary, this study applied the network pharmacology method to study complex network relationships regarding the multicomponent, multitarget and multichannel aspects of CKI for the treatment of GC. We predicted that CKI controls the growth and metastasis of GC cells by regulating vital targets such as CCND1, PIK3CA, and AKT1, as well as important related pathways. The results of the study preliminarily verified and predicted the molecular mechanism of CKI against GC but still need further experimental verification. The results provide a basis for further exploration of the mechanism of action and of CKI for the treatment of GC and provides a reference for the study of the more complex mechanism of action of this Chinese herbal compound.

## Supplementary information

**Additional files 1: Table S1.** CKI Compound.

**Additional files 2: Table S2.** Target of GC.

**Additional files 3: Table S3.** Target of CKI Compound.

## Data Availability

The datasets used and/or analyzed during the current study are available from the corresponding author upon reasonable request.

## Abbreviations

CCND1: Cyclin D1
CKI: Compound Kushen Injection
DAVID: Database for Annotation, Visualization and Integrated Discovery
ERK2: Extracellular signal-regulated kinase 2
FDR: False discovery rate
GC: Gastric cancer
GO: Genome Ontology
KEGG: Kyoto Encyclopedia of Genes and Genomes
MAPKs: Mitogen-activated protein kinases
OMIM: Online Mendelian Inheritance in Man
PDB: Protein Data Bank
PharmGKB: Pharmacogenomics Knowledge Base
PKB: Protein kinase B
PKD/PKI: Negative logarithm of the experimental dissociation/suppression constant
PPI network: Protein-protein interaction network
STRING: Search Tool for the Retrieval of Interacting Genes
TCM: Traditional Chinese medicine
TTD: Therapeutic Target Database
