# Supplementary material for: Study on the mechanisms of compound Kushen injection for the treatment of gastric cancer based on network pharmacology
Source: BMC Complement Med Ther. 2020 Jan 15;20:6. doi: 10.1186/s12906-019-2787-y (PMC7076865; doi:10.1186/s12906-019-2787-y)
Supplement: Supplementary file 1 — Additional files 1: Table S1. CKI Compound. [file 12906_2019_2787_MOESM1_ESM.docx]

**Table S1.** CKI Compound

| No. | Compound | No. | Compound |
| --- | --- | --- | --- |
| 1 | 5, 6-dehydrolupanine | 13 | matrine |
| 2 | 5α, 9α-hydroxymatrine | 14 | N-methylcytisine |
| 3 | 7, 11-dehydromatrine | 15 | oxymatrine |
| 4 | 9α-hydroxymatrine | 16 | oxysophocarpine |
| 5 | 9α-hydroxysophocarpine | 17 | oxysophoranol |
| 6 | adenine | 18 | oxysophoridine |
| 7 | baptifoline | 19 | piscidic acid |
| 8 | isomatrine | 20 | sophocarpine |
| 9 | isosophocarpine | 21 | sophoranol |
| 10 | lamprolobine | 22 | sophoridine |
| 11 | liriodendrin | 23 | trifolirhizin |
| 12 | macrozamin |  |  |
