# Supplementary material for: Study on the mechanisms of compound Kushen injection for the treatment of gastric cancer based on network pharmacology
Source: BMC Complement Med Ther. 2020 Jan 15;20:6. doi: 10.1186/s12906-019-2787-y (PMC7076865; doi:10.1186/s12906-019-2787-y)
Supplement: Supplementary file 2 — Additional files 2: Table S2. Target of GC. [file 12906_2019_2787_MOESM2_ESM.docx]

| Gene | Database | Gene | Database |
| --- | --- | --- | --- |
| TMP1 | TTD | PTGS2 | DigSEE |
| ERBB2 | TTD DigSEE | S100A8 | DigSEE |
| EGFR | TTD DigSEE PharmGKB | BCL2 | DigSEE |
| KDR | TTD | IL8 | DigSEE |
| SCN1A | TTD | BAX | DigSEE |
| RRM2 | TTD | CEACAM5 | DigSEE |
| GAST | TTD | MTHFR | DigSEE |
| TOP1 | TTD | MAPK1 | DigSEE |
| CD55 | TTD | MLH1 | DigSEE |
| GPR119 | TTD | CDKN1A | DigSEE |
| MET | TTD | CCND1 | DigSEE |
| KRAS | DisGeNET OMIM | AKT1 | DigSEE |
| APC | DisGeNET OMIM | TNF | DigSEE |
| IRF1 | DisGeNET OMIM | EGF | DigSEE |
| MUTYH | DisGeNET OMIM | JUN | DigSEE |
| CASP10 | DisGeNET OMIM | TYMS | DigSEE |
| CDH1 | DisGeNET DigSEE OMIM | NFKB1 | DigSEE |
| PIK3CA | DisGeNET | CASP3 | DigSEE |
| FGFR2 | DisGeNET OMIM | CTNNBIP1 | DigSEE |
| CHEK2 | DisGeNET | RUNX3 | DigSEE |
| NQO1 | PharmGKB | ABCB1 | DigSEE |
| TP53 | PharmGKB DigSEE | MYC | DigSEE |
| TMEM167AXRCC4 | PharmGKB | CDKN2A | DigSEE |
| VEGFA | PharmGKB DigSEE | AFP | DigSEE |
| IGFBP3 | PharmGKB | MMP2 | DigSEE |
| XRCC4 | PharmGKB | DPYD | DigSEE |
| NOS3 | PharmGKB | PIK3CA | OMIM |
| PON1 | PharmGKB | KLF6 | OMIM |
| ERCC1 | PharmGKB | IL1B | OMIM |
| CYP2A6 | PharmGKB | IL1RN | OMIM |

**TableS2.** Target of GC
