# Supplementary material for: Study on the mechanisms of compound Kushen injection for the treatment of gastric cancer based on network pharmacology
Source: BMC Complement Med Ther. 2020 Jan 15;20:6. doi: 10.1186/s12906-019-2787-y (PMC7076865; doi:10.1186/s12906-019-2787-y)
Supplement: Supplementary file 3 — Additional files 3: Table S3. Target of CKI Compound. [file 12906_2019_2787_MOESM3_ESM.docx]

| Compound | Gene Code |
| --- | --- |
| 9α-hydroxymatrine | MAPT |
| 9α-hydroxymatrine | MBNL1 |
| 9α-hydroxymatrine | MBNL2 |
| 9α-hydroxymatrine | MBNL3 |
| 9α-hydroxymatrine | AR |
| 9α-hydroxymatrine | HSD11B1 |
| 9α-hydroxymatrine | HSD11B1L |
| 9α-hydroxymatrine | BCHE |
| 9α-hydroxymatrine | ACHE |
| 9α-hydroxymatrine | PRKCG |
| 9α-hydroxymatrine | PRKCB |
| 9α-hydroxymatrine | PRKCA |
| 9α-hydroxymatrine | PRKCQ |
| 9α-hydroxymatrine | PRKCD |
| 9α-hydroxymatrine | SRD5A2 |
| adenine | ADORA2A |
| adenine | ADORA1 |
| adenine | ADORA2B |
| adenine | GDA |
| adenine | CDK2 |
| adenine | CCNE1 |
| adenine | TDP1 |
| adenine | O00408 |
| adenine | ADA |
| adenine | AHCY |
| adenine | AHCYL1 |
| adenine | AHCYL2 |
| adenine | HPRT1 |
| adenine | PTGS1 |
| adenine | MAPK3 |
| adenine | PRTFDC1 |
| baptifoline | CHRNA7 |
| baptifoline | CHRFAM7A |
| baptifoline | CHRNB2 |
| baptifoline | CHRNB4 |
| baptifoline | CHRNA3 |
| baptifoline | CHRNA4 |
| baptifoline | CHRNA5 |
| baptifoline | CHRNB3 |
| baptifoline | CHRNA2 |
| baptifoline | CHRNA6 |
| baptifoline | DRD2 |
| baptifoline | DRD4 |
| baptifoline | DRD3 |
| baptifoline | MBNL1 |
| isomatrine | MBNL1 |
| isomatrine | MBNL2 |
| isomatrine | MBNL3 |
| isomatrine | CHRNA7 |
| isomatrine | CHRFAM7A |
| isomatrine | CHRNB2 |
| isomatrine | CHRNA4 |
| isomatrine | CHRNB4 |
| isomatrine | CHRNA3 |
| isomatrine | HSD11B1 |
| isomatrine | HSD11B1L |
| isomatrine | MAPT |
| isomatrine | FAAH |
| isomatrine | BCHE |
| isomatrine | ACHE |
| isomatrine | SLC6A2 |
| isomatrine | SLC6A3 |
| lamprolobine | CHRNB2 |
| lamprolobine | CHRNA4 |
| lamprolobine | CHRNA7 |
| lamprolobine | CHRFAM7A |
| lamprolobine | MBNL1 |
| lamprolobine | MBNL2 |
| lamprolobine | MBNL3 |
| lamprolobine | CHRNB4 |
| lamprolobine | CHRNA3 |
| lamprolobine | HSD11B1 |
| lamprolobine | HSD11B1L |
| lamprolobine | BCHE |
| lamprolobine | ACHE |
| lamprolobine | CHRM1 |
| lamprolobine | MAPT |
| lamprolobine | CHRM2 |
| lamprolobine | CHRM4 |
| liriodendrin | ADORA1 |
| liriodendrin | ADORA2A |
| liriodendrin | ADORA2B |
| liriodendrin | PTPN2 |
| liriodendrin | PTPN1 |
| liriodendrin | MAPT |
| liriodendrin | TYR |
| liriodendrin | P17643 |
| liriodendrin | DCT |
| liriodendrin | SRD5A1 |
| liriodendrin | SRD5A2 |
| liriodendrin | SOAT2 |
| liriodendrin | SOAT1 |
| liriodendrin | HIF1A |
| liriodendrin | SLC5A1 |
| macrozamin | LGALS3 |
| macrozamin | CDK1 |
| macrozamin | CDK4 |
| macrozamin | CDK2 |
| macrozamin | CDK3 |
| macrozamin | CDK6 |
| macrozamin | MBNL1 |
| macrozamin | MBNL2 |
| macrozamin | MBNL3 |
| macrozamin | CA1 |
| macrozamin | CA2 |
| macrozamin | CA3 |
| macrozamin | P35218 |
| macrozamin | CA7 |
| macrozamin | CA13 |
| matrine | MBNL1 |
| matrine | MBNL2 |
| matrine | MBNL3 |
| matrine | CHRNA7 |
| matrine | CHRFAM7A |
| matrine | CHRNB2 |
| matrine | CHRNA4 |
| matrine | CHRNB4 |
| matrine | CHRNA3 |
| matrine | HSD11B1 |
| matrine | HSD11B1L |
| matrine | MAPT |
| matrine | FAAH |
| matrine | BCHE |
| matrine | ACHE |
| matrine | SLC6A2 |
| matrine | SLC6A3 |
| N-methylcytisine | CHRNA7 |
| N-methylcytisine | CHRFAM7A |
| N-methylcytisine | CHRNB2 |
| N-methylcytisine | CHRNB1 |
| N-methylcytisine | CHRND |
| N-methylcytisine | CHRNA1 |
| N-methylcytisine | CHRNG |
| N-methylcytisine | CHRNB4 |
| N-methylcytisine | CHRNA3 |
| N-methylcytisine | CHRNA4 |
| N-methylcytisine | CHRNA2 |
| N-methylcytisine | CHRNA5 |
| N-methylcytisine | CHRNB3 |
| N-methylcytisine | CHRNA6 |
| N-methylcytisine | DRD2 |
| N-methylcytisine | DRD4 |
| N-methylcytisine | DRD3 |
| oxymatrine | MBNL1 |
| oxymatrine | MBNL2 |
| oxymatrine | MBNL3 |
| oxymatrine | MMP1 |
| oxymatrine | MMP3 |
| oxymatrine | MMP8 |
| oxymatrine | MMP10 |
| oxymatrine | MMP27 |
| oxymatrine | CHRNA7 |
| oxymatrine | CHRFAM7A |
| oxymatrine | SRD5A1 |
| oxymatrine | SRD5A2 |
| oxymatrine | HSD11B1 |
| oxymatrine | HSD11B1L |
| oxymatrine | CHRNB2 |
| oxymatrine | CHRNA4 |
| oxysophocarpine | SRD5A1 |
| oxysophocarpine | SRD5A2 |
| oxysophocarpine | AR |
| oxysophocarpine | KCNH2 |
| oxysophocarpine | KCNH6 |
| oxysophocarpine | KCNH7 |
| oxysophocarpine | BCHE |
| oxysophocarpine | ACHE |
| oxysophocarpine | CTSL2 |
| oxysophocarpine | CTSL1 |
| oxysophocarpine | CTSS |
| oxysophocarpine | CTSK |
| oxysophocarpine | CHRNB2 |
| oxysophocarpine | CHRNA4 |
| oxysophocarpine | PGR |
| oxysophocarpine | ESR1 |
| piscidic acid | FOLH1 |
| piscidic acid | NAALADL1 |
| piscidic acid | NAALAD2 |
| piscidic acid | KDM4E |
| piscidic acid | KDM4A |
| piscidic acid | KDM4B |
| piscidic acid | KDM4D |
| piscidic acid | KDM4C |
| piscidic acid | CPA1 |
| piscidic acid | CPA3 |
| piscidic acid | CPB2 |
| piscidic acid | CPB1 |
| piscidic acid | CPA2 |
| piscidic acid | CPA5 |
| piscidic acid | CPA4 |
| sophocarpine | KCNH2 |
| sophocarpine | KCNH6 |
| sophocarpine | KCNH7 |
| sophocarpine | SRD5A2 |
| sophocarpine | SRD5A1 |
| sophocarpine | OPRM1 |
| sophocarpine | OPRD1 |
| sophocarpine | OPRK1 |
| sophocarpine | OPRL1 |
| sophocarpine | AR |
| sophocarpine | BCHE |
| sophocarpine | ACHE |
| sophocarpine | CHRNB2 |
| sophocarpine | CHRNA4 |
| sophocarpine | DRD2 |
| sophocarpine | Q14957 |
| sophoranol | BCHE |
| sophoranol | ACHE |
| sophoranol | PRKCG |
| sophoranol | PRKCB |
| sophoranol | PRKCA |
| sophoranol | PRKCQ |
| sophoranol | PRKCD |
| sophoranol | MBNL1 |
| sophoranol | MBNL2 |
| sophoranol | MBNL3 |
| sophoranol | MAPT |
| sophoranol | AR |
| sophoranol | CHRNB2 |
| sophoranol | CHRNA4 |
| sophoranol | CHRNA7 |
| sophoranol | CHRFAM7A |
| sophoridine | MBNL1 |
| sophoridine | MBNL2 |
| sophoridine | MBNL3 |
| sophoridine | CHRNA7 |
| sophoridine | CHRFAM7A |
| sophoridine | CHRNB2 |
| sophoridine | CHRNA4 |
| sophoridine | CHRNB4 |
| sophoridine | CHRNA3 |
| sophoridine | HSD11B1 |
| sophoridine | HSD11B1L |
| sophoridine | MAPT |
| sophoridine | FAAH |
| sophoridine | BCHE |
| sophoridine | ACHE |
| sophoridine | SLC6A2 |
| sophoridine | SLC6A3 |
| trifolirhizin | ADORA1 |
| trifolirhizin | CRYZ |
| trifolirhizin | CA1 |
| trifolirhizin | CA2 |
| trifolirhizin | CA3 |
| trifolirhizin | P35218 |
| trifolirhizin | CA7 |
| trifolirhizin | CA13 |
| trifolirhizin | Q9Y2D0 |
| trifolirhizin | CA12 |
| trifolirhizin | CA9 |
| trifolirhizin | CA14 |
| trifolirhizin | ADORA2A |
| trifolirhizin | ADORA2B |
| trifolirhizin | SLC5A1 |
| adenine | MTAP |
| adenine | PNP |
| adenine | APRT |
| adenine | ACACB |
| adenine | HPRT1 |
| adenine | PECR |
| adenine | ACP1 |
| adenine | ENSG00000264545 |
| adenine | ACACA |
| adenine | P2RY1 |
| matrine | PTEN |
| matrine | KCNH2 |
| matrine | CCND1 |
| matrine | MMP9 |
| oxymatrine | KCNH2 |
| oxymatrine | TLR4 |
| oxymatrine | TLR2 |
| adenine | APRT |
| adenine | ACACB |
| adenine | ACP1 |
| adenine | PECR |
| adenine | SRPK2 |
| adenine | MTAP |
| N-methylcytisine | CHRNA4 |
| N-methylcytisine | CHRNA7 |
| oxymatrine | CBX1 |
| oxymatrine | GNAS |
| adenine | ALOX5 |
| adenine | MAPK8 |
| adenine | CDK1 |
| adenine | EGFR |
| adenine | GSK3B |
| adenine | IKBKB |
| adenine | IGF1R |
| adenine | MAPK14 |
| adenine | MMP1 |
| adenine | MMP3 |
| adenine | MMP8 |
| adenine | MMP9 |
| adenine | MMP2 |
| adenine | PIK3CG |
| adenine | PRKCA |
| adenine | ERBB2 |
| adenine | ERBB4 |
| adenine | ROCK1 |
| adenine | AKT1 |
| adenine | AURKA |
| adenine | AURKB |
| adenine | BRAF |
| adenine | RAF1 |
| adenine | JAK3 |
| adenine | LCK |
| adenine | SYK |
| adenine | XDH |
| adenine | MAPK9 |
| adenine | PRKACA |
| adenine | MAP2K1 |
| adenine | PTK2 |
| adenine | HSP90AB1 |
| adenine | MAPK3 |
| adenine | MAPKAPK2 |
| adenine | MAP3K5 |
| adenine | MAPK1 |
| adenine | NFE2L2 |
| adenine | PIK3CA |
| adenine | PIK3CD |
| adenine | LMNA |
| adenine | PDK1 |
| adenine | AKT2 |
| adenine | PAK2 |
| adenine | PIM1 |
| adenine | RIPK2 |
| adenine | SGK1 |
| adenine | TARDBP |
| adenine | TSG101 |
| adenine | FES |
| adenine | ITK |

**TableS3.** Target of CKI Compound
